# Emerging adults facing the COVID-19 pandemic: emotion dysregulation, mentalizing, and psychological symptoms

**DOI:** 10.1007/s12144-022-03322-5

**Published:** 2022-06-21

**Authors:** Simone Charpentier Mora, Chiara Bastianoni, Donatella Cavanna, Fabiola Bizzi

**Affiliations:** Department of Educational Sciences, University of Genoa, Corso A. Podestà 2, 16121 Genoa, Italy

**Keywords:** Mentalizing, Emotion dysregulation, Psychological symptoms, COVID-19, Longitudinal study

## Abstract

Settled in the context of the COVID-19 pandemic, the present short-term longitudinal study aims to investigate the relation between emotion dysregulation, mentalizing (both certainty and uncertainty about mental states), and psychological symptoms in a sample of 83 emerging adults (M_age_ = 22.18 years, SD = 4.36) over a continuous period started with COVID-19 spreads. Results display significant positive associations between psychological symptoms and both emotion dysregulation and uncertainty about mental states, while an inverse association with certainty about mental states was found. A moderation model was also performed, showing a significant negative association between emotion dysregulation and psychological symptoms at low levels of uncertainty about mental states. Conversely, a marginally significant positive association occurs at high levels of uncertainty about mental states. In other words, the presence of individual impairments in perceiving one’s own/others mind may increase the negative consequences of emotion dysregulation on reported psychological symptoms. To sum up, our findings highlight the importance of considering mentalizing as a possible key factor for the promotion of emerging adults’ mental health also in the context of the ongoing COVID-19 pandemic.

## Introduction

A large body of theoretical work considers emotion regulation (ER) and mentalizing (operationalized as Reflective Functioning, RF) as health-promoting resources for the overall psychological functioning (Beauchaine & Cicchetti, 2019; Luyten et al., 2020). Differently, maladaptive forms of emotion regulation (i.e. emotion dysregulation, ED), together with uncertainty of one’s own and other’s mental states (e.g. Aldao et al., 2010; Bizzi et al., 2022), have been associated with psychological symptoms; specifically RF impairments are considered as effective buffers for a wide range of psychopathology in individuals exposed to trauma (Antonsen et al., 2015; Chiesa & Fonagy, 2014; Schwarzer et al., 2021).

Among the traumatic experiences that may impact individual and relational life, the COVID-19 pandemic is still considered as a highly stressful condition for most people which may lead to the so called “pandemic fatigue” (Haktanir et al., 2021; WHO, 2020), and it is also likely to represent a traumatic one for many others (Horesh & Brown, 2020). Social isolation, changes in routines and governmental restrictions may thus have affected subjective mental health (Alzueta et al., 2020; Boden et al., 2021) by reducing the range of strategies through which people usually regulate their emotions. As a consequence, people who in the past would present as very low risk, are now exposed to high levels of stress which increase their emotional vulnerability, with possible collapses in emotion regulation strategies and mentalizing abilities, as well as the emergence of psychological symptoms (Lassri & Desatnik, 2020). This could be the case of emerging adults that despite being less likely to get a severe COVID-19 infection, are involved in a developmental period characterized by search of autonomy and identity formation, and during which interpersonal relationships play a crucial role (Arnett, 2015; Li et al., 2021). Following this, the unpredictable changes determined by the COVID-19 pandemic have deeply threatened emerging adults’ overall mental health by increasing their perceived insecurity (Wen et al., 2022). In light of these considerations and based on the theoretical framework defined by the mentalizing approach to psychopathology (Luyten et al., 2020), the present longitudinal study aims to investigate the relation between emotion dysregulation (ED), RF (both Certainty and Uncertainty about mental states), and psychological symptoms (i.e. Global Severity Index, GSI) in emerging adults over the continuous period defined by the COVID-19 pandemic (from May 2020 to March 2021). Given the aforementioned studies (Aldao et al., 2010; Beauchaine & Cicchetti, 2019; Luyten et al., 2020), which highlighted a link between these variables, it was hypothesized that: (1) ED would be positive associated with GSI; (2) RF would be both negatively (Certainty about mental states) and positively (Uncertainty about mental states) associated with GSI; (3) RF would moderate the relationship between ED and GSI.

## Materials and Methods

### Design and Participants

We used a short-term longitudinal and correlational design. The sample consisted of 83 emerging adults (M_age_ = 22.18 years, SD = 4.36; 57.8% females; M_education_ = 14.87 years, SD = 2.32) drawn from the general population and recruited using a snowball sampling technique among university students in two different Italian cities located in the north and in the center of the country.

## Materials and Procedure

The research was approved by the ethics committee of the University of Genoa (protocol n. 41/2020). Data were collected online between May 2020 and March 2021 during the COVID-19 pandemic through a survey on three times. Time 1: first wave of COVID-19, May 2020; Time 2: second wave of COVID-19, October 2020; Time 3: third wave of COVID-19, March 2021. Study aims and information regarding anonymity and privacy were illustrated through a presentation letter sent to all the identified participants. They were next invited to provide an informed written consent before filling the following battery of self-report questionnaires.

At Time 1, we administrated the following instruments:

### Demographic Information:

a questionnaire created to evaluate age, sex, level of education and a single-item measure on a 5-point Likert scale (i.e. “How would you describe your psychological well-being before the COVID-19 pandemic?”) to assess psychological well-being in the pre-COVID-19 period.

### Difficulties in Emotion Regulation Scale

(DERS; Gratz & Roemer, 2004): a self-report questionnaire consisting of 36 items on a 5-point Likert scale, providing a total score of emotion regulation difficulties and six separated scores related to the subscales of the instrument: Non-Acceptance, Awareness, Clarity, Strategies, Goals and Impulse. In the current study, we used DERS total score who showed good internal consistency (Cronbach’s α = 0.83).

At Time 2, we administered the following instrument:

### Reflective Functioning Questionnaire

(RFQ; Fonagy et al., 2016): a self-report questionnaire consisting of 54 items on a 7-point Likert scale to evaluate mentalizing abilities by measuring the degree of Certainty (RFQc) and Uncertainty (RFQu) with which individuals utilize mental state information to understand their own and others’ behavior. In the present study, the instrument showed good internal consistency (Cronbach’s α: RFQc = 0.88; RFQu = 0.87).

At Time 3, we administered the following instrument:

### Symptom Checklist-90-Revised

(SCL-90-R; Derogatis & Cleary, 1977): a self-report questionnaire consisting of 90 items on a 5-point Likert scale providing a description of psychological symptomatology through 9 subscales and a Global Severity Index (GSI) which summarizes the previous ones. In the current study, we used GSI who showed good internal consistency (Cronbach’s α = 0.98).

## Data Analyses

Sex differences were evaluated using a *t*-test for independent samples. Pearson’s correlations between study’s variables were next conducted to test first two hypotheses. For testing the third hypothesis, we used PROCESS macro for SPSS (Hayes, 2018) with 5.000 bias-corrected bootstrap samples. We considered the effects significant at *p* value < 0.05 and when the 95% confidence interval (CI) did not include 0. There was no evidence of multicollinearity (tolerance values > 0.05 and VIF values < 2 for all variables) and all variables were normally distributed according to the Shapiro-Wilk test (W = 0.98, *p* = .38) evaluated on the residuals of our regression model.

## Results

### Descriptive Statistics and Preliminary Analyses

Potential covariates (i.e. sex, age, level of education and psychological well-being in a pre-COVID-19 period) were examined in relation to predictors and outcome variables. Age and level of education were not significantly associated with any of the study variables, while a significant negative correlation between psychological well-being in a pre-COVID-19 period and GSI (*r* = -.47, *p* < .01) was found, together with a significant association between sex and GSI (*t*_(50) _= -2.50, *p* = .016, d = 0.71). Thus, sex and psychological well-being in a pre-COVID-19 period were inserted as covariates in subsequent models. Descriptive statistics and correlations among study variables are reported in Table 1.

**Table 1:** Descriptive statistics and correlations among study variables

	2.	3.	4.	5.	6.	7.	M (SD)
1. Age	0.32**	− 0.10	− 0.13	− 0.01	− 0.14	− 0.01	26.18 (4.36)
2. Level of education		0.06	− 0.12	− 0.23	− 0.14	− 0.09	14.87 (2.32)
3. Psychological well-being			− 0.14	− 0.21	− 0.04	− 0.47**	3.89 (0.80)
4. DERS total score				0.39**	− 0.33**	0.31*	85.09 (18.18)
5. RFQu					− 0.36**	0.41**	11.25 (9.43)
6. RFQc						− 0.33*	20.00 (11.87)
7. GSI							0.72 (0.54)

*p < .05, **p < .01

## Regression Analyses

**Fig. 1:**
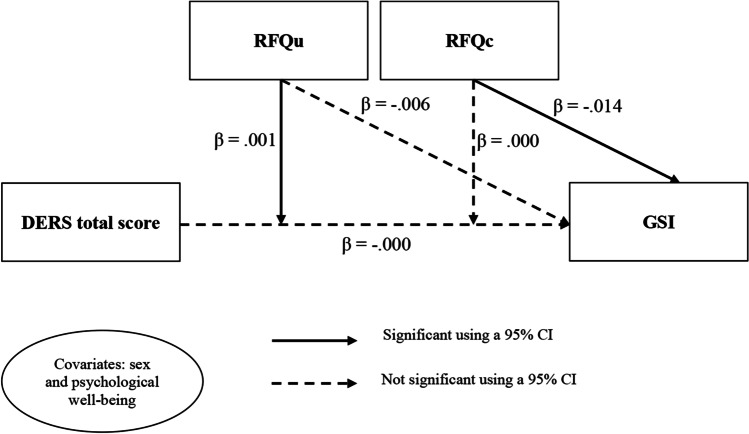
Moderating effect of RF on the link between ED and GSI Note. DERS total score = Emotion Dysregulation Total Score; RFQu = Uncertainty about Mental States; RFQc = Certainty about Mental States; GSI = Global Severity Index

To test whether DERS total score, RFQc and RFQu predict GSI correlation analyses and a moderated regression analysis were performed. Firstly, both DERS total score (*r* = .31, *p* = .03), RFQc (*r* = -.33, *p* = .02) and RFQu (*r* = .41, *p* < .01) were significantly associated with GSI. Secondly, we explored the role of DERS total score and its interaction with RFQc and RFQu in predicting GSI. In this model, we inserted GSI as dependent variable, DERS total score as independent variable, RFQc and RFQu as moderator variables. Sex and psychological well-being in a pre-COVID-19 period were included as covariates. The results indicated that sex (*β* = 0.262, *SE* = 0.114, *p* = .03, CI [0.032, 0.491]), psychological well-being (*β* = -0.255, *SE* = 0.079, *p* < .01, CI [-0.414, -0.095]), RFQc (*β* = -0.014, *SE* = 0.006, *p* = .03, CI [-0.027, -0.001]), as well as the interaction between RFQu and DERS total score (∆R^2^ = 0.11, *β* = 0.001, *SE* = 0.000, CI [0.000, 0.002]) predicted GSI. The model explained 55% of the variance in GSI. As presented in Fig. 1, a negative association between DERS total score and GSI occurred when RFQu was low (*β* = -0.016, *SE* = 0.006, *t*_(43) _= -2.56, *p* = .01, CI [-0.028, -0.003]) and a marginally positive association between DERS total score and GSI occurred when RFQu was high (*β* = 0.001, *SE* = 0.005, *t*_(43)_ = 1.97, *p* = .05, CI [-0.000, 0.020]) while there was no association when the participants had a medium level of RFQu (*β* = -0.001, *SE* = 0.004, *t*_(43)_ = -0.23, *p* = .82, CI [-0.008, 0.007]). Finally, the moderating effect of RFQc on the link between DERS total score and GSI was not significant (∆R^2^ = 0.01, *β* = 0.000, *SE* = 0.000, CI [-0.000, 0.001]). The moderation model is presented in Fig. 1.

## Discussion

The present study aimed to investigate possible relations between ED, RF (both Certainty and Uncertainty about mental states), and GSI (psychological symptoms) at different times of COVID-19 pandemic within an Italian sample of emerging adults. In line with previous studies addressing the relation among similar variables (Aldao et al., 2010; Beauchaine & Cicchetti, 2019; Luyten et al., 2020), correlation analyses showed significant positive associations between GSI and both ED and uncertainty about mental states (RFQu), while an inverse association with certainty about mental states (RFQc) was found, thus confirming our first two hypotheses. Regarding our third hypothesis, the performed model displayed a significant moderating effect of RFQu – but not of RFQc – in the link between ED and GSI, so that at high levels of RFQu, reflecting an almost complete lack of knowledge about mental states, a marginally significant positive relation between ED and GSI was found, while at low levels of RFQu a significant negative relation between ED and GSI emerged. Settled in the context of the COVID-19 pandemic, which has been defined as an epidemiological and psychological crisis (APA, 2020), these results highlight that the stressful conditions related to the social isolation imposed by the lockdown measures during the first wave of the COVID-19 pandemic may have impacted emerging adults’ ER strategies, thus enhancing mental health vulnerabilities over the third wave of the COVID-19 pandemic (Lassri & Desatnik, 2020). Moreover, the reported moderation role acted by RFQu – and not by RFQc, which nevertheless showed a direct effect on GSI – may underline the central role performed by RF in influencing reported psychological symptoms, thus emphasizing possible different functions displayed by these two dimensions (Fonagy et al., 2016). In that sense, the presence of substantial individual impairments in perceiving the complex models of one’s own/others mind may increase the negative consequences of ED on reported psychological symptoms. These aspects could be particularly important for emerging adult individuals who are experiencing this specific transitional period characterized by issues and oscillations in the sense of autonomy and belongingness toward the broad relational environment outside the family system. All these challenges could be affected by the perturbing period represented by the spread of COVID-19 pandemic and its ongoing consequences, determining a complex interplay between developmental tasks and environmental conditions that may increase the risk for the onset of psychological symptoms (Germani et al., 2020; Marchini et al., 2021; Wen et al., 2022). Despite its interesting findings, our study also includes some important limitations, such as the limited sample size, the use of a single-item measure to control psychological well-being in the pre-COVID-19 period and the use of self-report instruments instead of more detailed measures to assess mentalizing abilities. Despite these, the longitudinal design employed for this study offers possible suggestions for future contributions, addressing the importance of strengthening mentalizing abilities in emerging adults for improving their psychological well-being during an epidemiological and psychological crisis such as the COVID-19 pandemic.

## Clinical Implications and Future Directions

The findings of the current study entail several clinical implications. Firstly, they stress the importance of considering ED as a key process in clinical interventions aimed to help emerging adults in developing adaptive emotion regulation strategies as protective factors for their mental health during the ongoing COVID-19 pandemic. Moreover, the different role played by the two components of RF suggests the importance to give greater attention to the different mentalizing dimensions in order to improve preventive and clinical interventions. Following these directions, the main goal should be to foster an attitude of interest and curiosity towards one’s own and others’ mental states and also to promote the development of emotion regulation strategies that lead individuals to be flexibly capable of finding a source of reassurance in both themselves and others. In broader terms it can be concluded that mentalizing may play a fundamental role in the promotion of emerging adults’ mental health. The capability to reflect upon their own and others’ inner mental world should therefore protect them from the negative consequences of stress-affected emotion dysregulation in the context of the COVID-19 pandemic. Promoting a reengaging of mentalizing abilities – also at a later stage after the pandemic spread – may thus result in an improvement of community’s mental health conditions. In light of these, mentalizing should be considered in future studies as a central transtheoretical and transdiagnostic concept, useful to comprehend and intervene on mental health vulnerabilities during this COVID-19 era.

## Data Availability

The dataset analyzed during the current study is available from the corresponding author on reasonable request.
